# Cost of Illness, Quality of Life, and Work Outcomes in Active Ankylosing Spondylitis Patients Treated With Adalimumab in China

**DOI:** 10.3389/fpubh.2020.602334

**Published:** 2020-11-24

**Authors:** Liudan Tu, Ya Xie, Zetao Liao, Yutong Jiang, Qing Lv, Shuangyan Cao, Qiujing Wei, Jieruo Gu

**Affiliations:** Department of Rheumatology, Third Affiliated Hospital of Sun Yat-sen University, Guangzhou, China

**Keywords:** ankylosing spondylitis, work outcomes, cost of illness, quality of life, adalimumab

## Abstract

**Objectives:** To access the cost of illness, quality of life and work limitation in active ankylosing spondylitis (AS) patients using adalimumab in China.

**Methods:** A prospective study was performed in 91 patients with active AS in China. Adult patients (aged ≥ 18 years) fulfilled the 1984 New York modified criteria of AS with the Bath Ankylosing Spondylitis Disease Activity Index ≥ 4 were enrolled. All participants received adalimumab (40 mg per 2 weeks) therapy and completed questionnaires about disease characteristics, quality of life and cost. Only patients with pay-work completed the Work Limitation Questionnaire and Work productivity and activity impairment questionnaire in AS. Factors associated with work outcomes were evaluated.

**Results:** A total of 91 patients with mean age of 30 years old (87.8% males) and mean disease duration of 10 years received adalimumab treatment for 24 weeks. The annual estimated cost of each patient was $37581.41 while the direct cost accounted for 84.6%. Seventy-eight percent of patients have a paid job with average work productivity loss of 0.28 measured by work limitation questionnaire, absenteeism and presenteeism were 10.22 and 43.86%, respectively, with a mean work productivity loss of 47.92% measured by Work productivity and activity impairment questionnaire in AS. Patients experienced significantly greater improvements after adalimumab treatment in presenteeism, absenteeism, work productivity, and quality of life.

**Conclusions:** The cost of AS patients with adalimumab therapy was high in China. Disease activity, physical function, quality of life, and work outcomes improved significantly after therapy.

## Introduction

Ankylosing spondylitis (AS) is a chronic inflammatory disease characterized with low back pain, morning stiffness, peripheral joints and extra-articular manifestations. With a prevalence of 0.3% (1) and young age onset in China, AS may lead to limited physical function, impaired quality of life and increased economic burden for society. Besides, more attention of the person's career, family and social life have been rose from both patients and doctors' perspective (2).

Work ability plays an important role in people's daily life and is a core component of family income. International guidelines from the Canadian Rheumatology Association and the Spondyloarthritis Research Consortium of Canada recommended that work activities should be included as part of disease monitoring (3). It has been reported that withdrawal from work and work instability were more common in AS patients than general population (4), even non-radiographic spondyloarthritis (SpA) patients experienced similar disease burden as (5). In China, spark researches focus on the work ability and indirect cost of AS (6), although many studies indicated that indirect cost including cost caused by work inability may contribute to a large scale of total cost (7).

The 2013 China Health Insurance Research Association (CHIRA) database including 1,299 patients with AS reported that only 4.5% received biologic agents with a mean direct medical cost of 14539RMB (8), indicating significant barrier for patients access to biologic agents because of higher cost and hospital-based reimbursement policy in China. Treatment with adalimumab in AS patients has been proved to be effective in symptom release, disease activity and functional remission in several studies (9, 10). Meanwhile, the improvements of quality of life and work outcomes (11, 12) have been observed after treated with adalimumab. However, the impact of adalimumab treatment on disease burden and work outcomes is rarely reported in Chinese AS patients. In this study, we aim to estimate the cost of illness, work ability, quality of life and related factors among AS patients treated with adalimumab in China.

## Methods

### Study Design and Patients

This study is a prospective, open-label, post-authorization, observational study in AS patients, focusing on the effects of adalimumab on work productivity, and quality of life. Consecutive patients with active AS were enrolled between July 2017 and Jan 2018 at the Rheumatology clinic of third affiliated hospital of Sun Yat-sen University of China. Inclusion criteria includes fulfilling the 1984 modified New York criteria for AS, disease activity measured by Bath Ankylosing Spondylitis Disease Activity Index (BASDAI) ≥4 after treated with at least 4 weeks' full dose of non-steroidal anti-inflammatory drugs (NSAIDs). Exclusion criteria includes tumor, other rheumatic diseases and serious infections. This study was approved by the ethics board of third affiliated hospital of Sun Yat-sen University and all patients provided written informed consent before participation in this study. Patients received adalimumab 40 mg per 2 weeks for 24 weeks. Assessments including disease activity, physical function, work productivity and quality of life were performed at baseline, week 12 and 24.

### Socio-Demographic and Clinical Characteristics

Socio-demographic characteristics including age, sex, marital status, household monthly income, employment, and education were collected. Clinical features such as disease duration, delayed diagnosis time, family history, BASDAI, Ankylosing Spondylitis Disease Activity Score (ASDAS), use of medications, Bath Ankylosing Spondylitis Functional Index (BASFI), Bath Ankylosing Spondylitis Metrology Index (BASMI), lab examinations including C reactive protein (CRP), and erythrocyte sedimentation rate (ESR) were recorded through face-to-face interview surveys and medical charts.

### Cost of Illness (COI)

Direct cost and indirect cost were calculated to evaluate the COI of AS patients from societal perspective. Direct medical cost included inpatient cost, outpatient attendance, medication usage, examinations and physiotherapy while direct non-medical cost included transportation fees and paid helper for household. Indirect cost included unemployment, productivity loss from work, sick leave and early retirement due to disease. All cost in this study were presented with 2017 Chinese currency Renminbi (RMB) and exchanged for US dollar at the rate of 1: 0.15.

### Work Outcomes

The Work limitation questionnaire (WLQ) (13) is a 25-items questionnaire exploring the degree of limitations experienced due to chronic health problems. Four scales including time management, physical demands, mental-interpersonal and output demands scales are calculated, and each range from 0 (limited none of the time)-100 (limited all of the time). Besides, a total WLQ index score can be calculated and converted into an estimate of productivity loss.

Work productivity and activity impairment questionnaire in AS (WPAI:SpA) (14) is a self-administered questionnaire for assessing the impact of disease on productivity during the previous 7 days. Four scores are derived including percentage of absenteeism (percentage work time missed because of problem), percentage of presenteeism (percentage impairment while working because of problem), an overall work impairment score (percentage overall work impairment because of problem), and percentage of impairment in activities performed outside of work. Higher scores indicate poorer work productivity and greater activity impairment due to AS.

### Health-Related Quality of Life (QOL)

EuroQol-5D (EQ-5D) and Ankylosing Spondylitis Quality of Life (ASQoL) were used to measure QOL of patients based on face-to-face interview. The EQ-5D (15) comprises of five dimensions including mobility, self-care, usual activities, pain/discomfort and anxiety/depression, and a visual analog scale (VAS) ranging from 0 (worst imaginable health) to 100 (best imaginable health) was used to rate health status that day. A health-state utility is calculated using the Chinese-specific values set and ranged from −0.39 (the worst health state) to 1 (full health) (16). The mean minimally important difference (MID) for the EQ-5D was reported to be 0.074 (17). ASQoL is an 18-items disease specific questionnaire related to symptoms, functions and disease-related concerns in AS patients (18). A total score was calculated and ranged from 0 to 18, which higher score indicates poor QOL.

### Statistical Analysis

Quantitative data were summarized as mean and SD for normal distributions. Summary statistics such as frequency and percentage were used for categorical variables. *T-*test was used to evaluate the change of costs and QOL between baseline and treatment. Regression analysis including univariable and multivariable generalized linear regressions was used to test the effect of related factors on presenteeism, absenteeism, and work productivity loss. Variables with *P* < 0.1 in the univariable linear regressions were included in the subsequent multivariable regression analyses. Factors with *P* < 0.05 in the multivariable regression were considered statistically significant. Results were reported as regression coefficients with 95% confidence interval. All tests were two-sided and *P* < 0.05 was considered statistically significant. Stata version 12.0 was used to perform statistical analyses.

## Results

### Baseline Characteristics

A total of 91 patients were included during Jan 2017 to Jun 2018 in third affiliated hospital of Sun Yat-sen University. The average age of patients was 30.6 years with a mean disease duration of 10 years, and 87.8% of them were male. The mean BASDAI and BASFI score were 5.31 ± 1.02 and 4.23 ± 1.92, respectively. Quality of life (QOL) measured with EQ-5D and ASQoL was 0.58 and 9.1, respectively (demographics of this sample are presented in Table 1). The mean (SD) activity impairment of all patients due to ill-health was 48.57% (22.02%).

**Table 1:** Baseline socio-demographic and clinical characteristics of active ankylosing spondylitis patients treated with adalimumab (*n* = 91).

**Characteristics**	**Mean (SD)**
Age (year)	30.58 (7.76)
Sex (male), n (%)	79 (87.78%)
Marital status, n (%)	
Single	44 (48.35%)
Married	46 (50.55%)
Divorced	1 (1.1%)
Education, n (%)	
Middle school or less	25 (27.47%)
High school	36 (39.56%)
College or more	30 (32.97%)
Employment, n (%)	71 (78.02%)
Work productivity loss (0–1)	0.28 (0.28)
WAPI:SpA	
Absenteeism	10.22% (19.44%)
Presenteeism	43.86% (22.48%)
Work productivity loss	47.92% (25.81%)
Activity impairment	48.57% (22.02%)
Disease duration (year)	9.99 (6.93)
Delayed diagnosis time (year)	3.62 (4.69)
HLA-B27 positive, n (%)	78 (85.71%)
Positive family history, n (%)	33 (36.26%)
BASDAI (0–10)	5.31 (1.02)
BASFI (0–10)	4.23 (1.92)
BASMI (0–10)	2.98 (2.47)
ASDAS-CRP	3.54 (0.80)
ASQoL	9.1 (3.81)
EQ-5D	0.58 (0.22)
CRP	23.89 (19.82)
ESR	29.08 (21.90)
Biologic agents used before, n (%)	56 (61.54%)

*SD, Standard Deviation; WPAI:SpA, Work productivity and activity impairment questionnaire in AS; HLA-B27, Human leukocyte antigen-B27; BASDAI, Bath Ankylosing Spondylitis Disease Activity Index; BASFI, Bath Ankylosing Spondylitis Functional Index; BASMI, Bath Ankylosing Spondylitis Metrology Index; ASDAS, Ankylosing Spondylitis Disease Activity Score; ASQoL, Ankylosing Spondylitis Quality of Life; EQ-5D, EuroQol-5 Dimensions; CRP, C-Reactive Protein; ESR, Erythrocyte Sedimentation Rate*.

### Cost of Illness

The mean COI per patient per year was 37581.41 dollars. Of the total, the percentage of direct cost was 84.6% and most of them amounted to adalimumab treatment. Of the indirect cost, productivity loss accounts for 52.7% (Table 2). The mean COI per patient per year to improve one unit of QOL/BASDAI/ASDAS is 42835/16753.68/4742.59 dollars, respectively.

**Table 2:** Direct and indirect cost of AS patients using adalimumab in China (dollar).

	**Mean (SD)**	**% of total cost**
Direct cost	31794.57 (2396.79)	84.60%
Inpatient cost	22.79 (166.62)	0.06%
Outpatient attendance	4.57 (17.69)	0.01%
Medications	27685.39 (304.84)	73.67%
Examinations	991.86 (339.87)	2.64%
Physiotherapy	10.59 (30.09)	0.03%
Non-medical cost	3079.37 (2300.89)	8.19%
Indirect cost	5786.84 (7005.23)	15.39%
Productivity loss	3049.64 (3065.87)	8.11%
Sick leave	2737.19 (6429.79)	7.28%
Total cost per year	37581.41 (8276.86)	100%

*SD, Standard Deviation*.

### Improvements in Disease Activity, Quality of Life, and Work Status After Adalimumab Treatment

**Figure 1:**
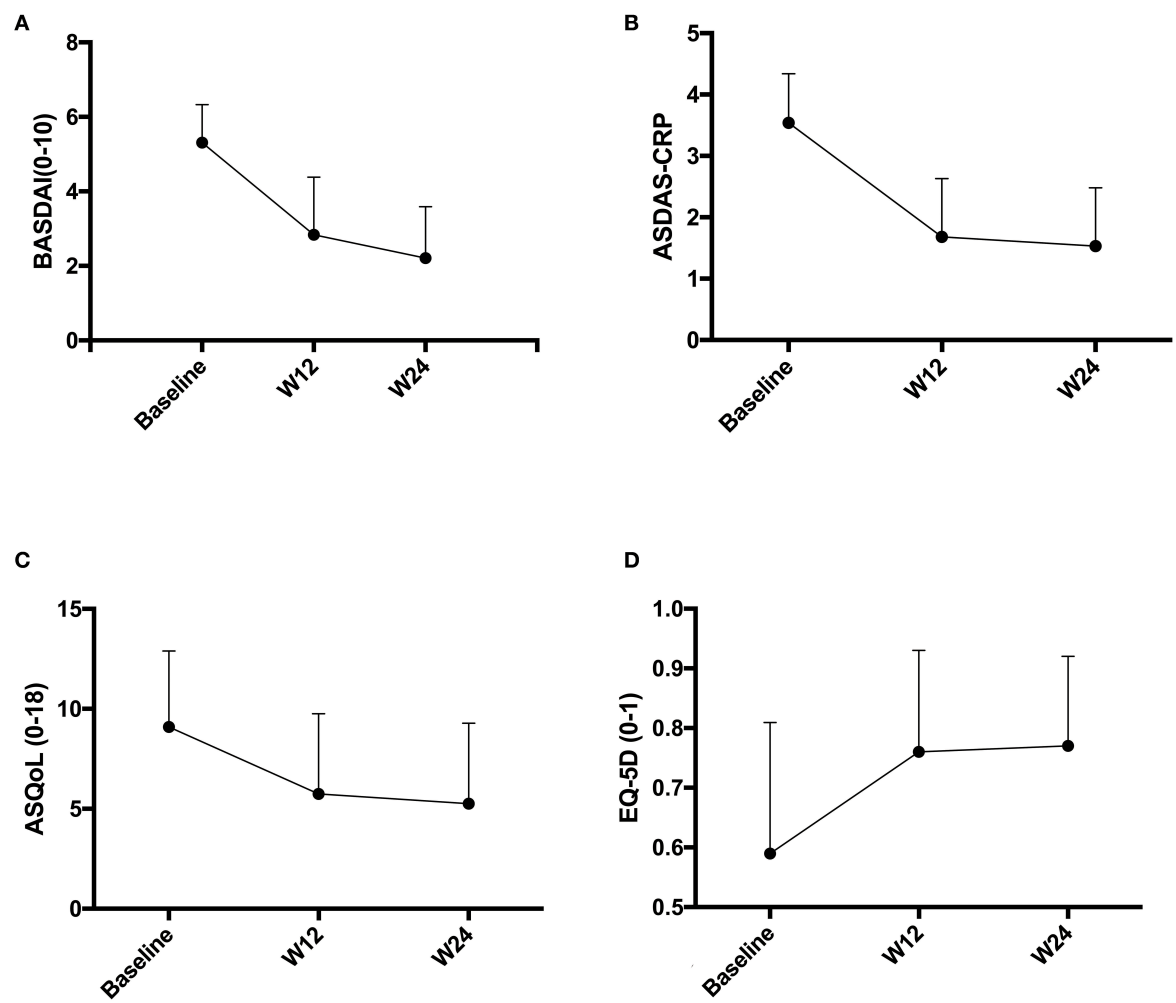
**(A–D)** Disease activity and quality of life change before and after adalimumab treatment. BASDAI, Bath Ankylosing Spondylitis Disease Activity Index; ASDAS, Ankylosing Spondylitis Disease Activity Score; ASQoL, Ankylosing Spondylitis Quality of Life; EQ-5D, EuroQol-5 Dimensions.

**Figure 2:**
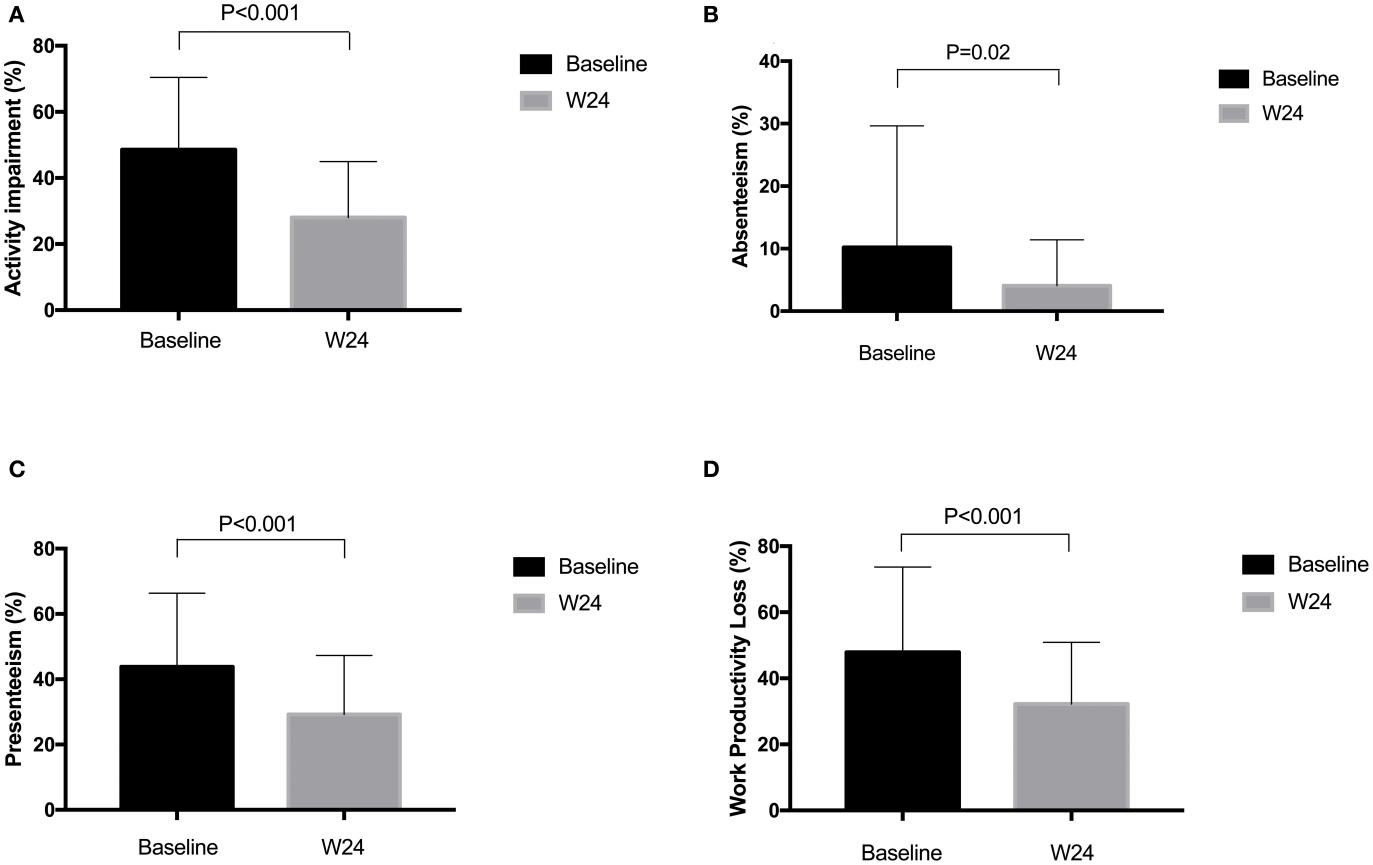
**(A–D)** Change of WPAI:SpA scores before and after adalimumab treatment. WPAI:SpA, Work productivity and activity impairment questionnaire in AS.

The improvements in BASDAI and ASDAS from baseline to week 24 were from 5.31 to 2.21 and from 3.54 to 1.53, respectively, in AS patients treated with adalimumab. Reduction of activity impairment was observed by study end in all patients. There were significant differences in change of ASQoL (change, 3.89 [95%CI, 3.06 to 4.71]; *P* < 0.0001) and EQ-5D (change, −0.19 [95%CI, −0.24 to −0.31]; *P* < 0.0001) scores from baseline and 24 weeks (Figure 1). Significant differences were found in work outcomes including absenteeism, presenteeism and work productivity loss at week 24 when compared with baseline (Figure 2).

### Work Outcomes and Related Factors at Baseline

Of the total 91 patients, 78.02% (*n* = 71) of patients were employed, mean (SD) work productivity loss measured by WLQ was 0.28 (0.28) due to disease. By using WAPI:SpA, mean (SD) absenteeism, presenteeism and work productivity loss were 10.22% (19.44%), 43.86% (22.48%), and 47.92% (25.81%), respectively.

In univariable analysis, age, marriage, education, ASDAS, QOL, CRP and ESR were associated with work productivity loss measured by WLQ at baseline. While only age (β: −0.01, *P* = 0.049) and education (β: −0.1, *P* = 0.03) remained significant in multivariable analysis. Table 3 show factors associated with WPAI scores at baseline. Disease duration was negatively associated with absenteeism (β: −0.71, *P* = 0.03), presenteeism (β: −0.77, *P* =0.04) and work productivity loss (β: −0.77, *P* = 0.02) in univariable analysis. These associations with absenteeism (β: −0.72, *P* = 0.03), presenteeism (β: −0.92, *P* = 0.04) were significant in multivariable analysis. BASFI was positively associated with activity impairment (β: 7.68, *P* < 0.01), presenteeism (β: 6.28, *P* < 0.01) and work productivity loss (β: 6.28, *P* < 0.01) in univariable analysis. In multivariable analysis, the associations with activity impairment (β: 4.94, *P* < 0.01), presenteeism (β: 4.63, *P* < 0.01), and work productivity loss (β: 5.35, *P* < 0.01) remained statistically significant. ASQoL was positively associated with activity impairment (β: 3.06, *P* < 0.01; β: 1.77, *P* < 0.01), presenteeism (β: 2.83, *P* < 0.01; β: 2.0, *P* < 0.01), and work productivity loss (β: 3.19, *P* < 0.01; β: 2.33, *P* < 0.01) in both univariable and multivariable analysis, respectively.

**Table 3:** Clinical characteristics associated with WPAI:SpA scores in AS patients at baseline.

	**Activity impairment**		**Absenteeism**		**Presenteeism**		**work productivity loss**	
	**Univariable β (95% CI)**	**Multivariable β (95% CI)**	**Univariable β (95% CI)**	**Multivariable β (95% CI)**	**Univariable β (95% CI)**	**Multivariable β (95% CI)**	**Univariable β (95% CI)**	**Multivariable β (95% CI)**
Age	−0.11 (−0.82, 0.59)	NA	−0.48 (−1.1, 0.15)	NA	−0.69 (−1.40, 0.01)	−0.27 (−1.07, 0.53)	−0.69 (−1.40, 0.01)	−0.58 (−1.54, 0.37)
Marriage	−2.65 (−12.67, 7.36)	NA	−6.26 (−15.14, 2.61)	NA	−3.73 (−13.98, 6.51)	NA	−3.74 (−13.98, 6.51)	NA
Education	−0.41 (−7.2, 6.37)	NA	3.5 (−2.6, 9.61)	NA	−0.88 (−7.83, 6.07)	NA	−0.88 (−7.83, 6.07)	NA
Disease duration	−0.002 (−0.71, 0.70)	NA	−0.71 (−1.35, −0.07)	**−0.72 (−1.37**, **−0.08)**	−0.77 (−1.49, −0.04)	**−0.92 (−1.77**, **−0.06)**	−0.77 (−1.49, −0.04)	−0.90 (−1.93, 0.12)
BASDAI	8.98 (4.19, 13.78)	2.08 (−4.12, 8.28)	3.56 (−1.04, 8.17)	NA	5.68 (0.47, 10.88)	−2.90 (−9.29, 3.48)	5.68 (0.47, 10.88)	−1.18 (−8.70, 6.33)
BASFI	7.68 (5.49, 9.87)	**4.94 (1.69, 8.19)**	1.49 (−1.06, 4.06)	NA	6.28 (3.76, 8.79)	**4.63 (1.28, 7.98)**	6.28 (3.76, 8.79)	**5.35 (1.48, 9.22)**
BASMI	3.30 (1.31, 5.28)	−0.52 (−2.72, 1.68)	−0.29 (−2.22, 1.64)	NA	1.22 (−0.95, 3.39)	NA	1.21 (−0.95, 3.38)	NA
ASDAS	13.45 (7.49, 19.41)	0.25 (−9.52, 10.01)	1.28 (−4.81, 7.36)	NA	8.94 (2.33, 15.54)	1.70 (−6.67, 10.08)	8.94 (2.33, 15.55)	−3.09 (−13.29, 7.10)
ASQOL	3.05 (1.87, 4.24)	**1.76 (0.52, 3.02)**	1.02 (−0.19, 2.23)	0.94 (−0.40, 2.28)	2.83 (1.57, 4.09)	**1.99 (0.65, 3.35)**	2.83 (1.57, 4.09)	**2.33 (0.80, 3.86)**
EQ-5D	−37.21 (−60.95, −13.48)	−2.25 (−29.98, 25.48)	−19.55 (−41.49, 2.39)	−8.82 (−32.87, 15.21)	−37.46 (−61.84, −13.08)	−5.92 (−36.12, 24.27)	−37.46 (−61.84, −13.08)	−4.62 (−39.27, 30.02)
CRP	0.34 (0.05, 0.62)	0.26 (−0.13, 0.66)	−0.002 (−0.27, 0.27)	NA	0.21 (−0.09, 0.51)	NA	0.21 (−0.09, 0.51)	NA
ESR	0.2 (−0.03, 0.44)	−0.07 (−0.31, 0.18)	0.12 (−0.09, 0.34)	NA	0.22 (−0.02, 0.46)	0.007 (−0.24, 0.26)	0.22 (−0.02, 0.46)	0.14 (−0.16, 0.43)

*CI, Confidence Interval; NA, Not Available (only variables with P < 0.1 in the univariable linear regressions were included in the subsequent multivariable regression analyses); WPAI:SpA, Work productivity and activity impairment questionnaire in AS; BASDAI, Bath Ankylosing Spondylitis Disease Activity Index; BASFI, Bath Ankylosing Spondylitis Functional Index; BASMI, Bath Ankylosing Spondylitis Metrology Index; ASDAS, Ankylosing Spondylitis Disease Activity Score; ASQoL, Ankylosing Spondylitis Quality of Life; EQ-5D, EuroQol-5 Dimensions; CRP, C-Reactive Protein; ESR, Erythrocyte Sedimentation Rate. Bold values are significant difference*.

## Discussion

In this study, marked improvements were observed in symptoms and physical function after adalimumab therapy, as well as QOL and work outcomes. These findings are consistent with other studies reporting improvement on QOL and work outcomes in AS patients treated with biological therapy (12, 19, 20). Besides, we found that disease duration, BASFI and ASQoL were associated with presenteeism. Disease duration was associated with absenteeism. Work productivity loss was related to age, education, BASFI and ASQoL. The strength of current study includes being a prospective study to evaluate cost of illness and work outcomes after adalimumab treatment of AS patients in China. Besides, this is the first study to explore factors associated with work outcomes in Chinese AS patients.

AS-related healthcare cost varied in different countries and it is difficult to compare work-related cost beyond the country of origin. In Brazil, a population-based cohort study reported 78% of AS patients initiating treatment with anti-TNF drugs (free of charge) with a median monthly cost per capita of $1650 (21). An UK research reported that the total cost of AS was estimated at €19016 per patient per year and the majority of the cost was a result of work-related costs (7). In this study, a mean COI per patient per year of $37581.41 were estimated with most of it accounted to adalimumab treatment, which may be because patients enrolled in this study had high disease activity and the fee of adalimumab was not covered by government insurance. When compared with a GDP per capita of $8800 in 2017 of China, the annual total cost of Chinese AS patients treated with adalimumab was still huge. Effective treatment can reduce disease severity, increase physical function and quality of life, improve work capacity and productivity, thus in return alleviating economic burden in the long term. Previous studies conducted in Europe (12) and Australia (22) showed decreased healthcare resource utilization and increased labor force participation rate after adalimumab therapy. In this study, improvement of work outcomes was observed after adalimumab treatment while no significant difference was found in total cost and indirect cost, indicating that it may take time to observe the long-term benefit.

Impact on work productivity in AS patients has drawn attention of researchers and doctors all around the world. A three-times more likelihood to withdraw from work and an employment rates varying from 55 to 89% in different countries in AS patients had been reported in several studies (23, 24). Positive effect of biological therapy on work outcomes have been proven in AS and axial SpA patients (11, 12, 19, 20). Study conducted in Europe reported that presenteeism decreased from 56.6 to 20.1%, absenteeism decreased from 15.6 to 6.4% and total work productivity impairment decreased from 59.9 to 22.1% after 1 year's adalimumab treatment (11). In this study, similar results were found that presenteeism decreased from 43.9 to 29.2%, absenteeism decreased from 10.2 to 4.1% and total work productivity impairment decreased from 47.9 to 32.2% after 6 months' therapy. In another review (20), no significant difference was found in absenteeism after biologic treatment. Absenteeism is a late stage in terms of work impairment that is not reversed by biological therapy alone but likely also to be influenced by contextual factors. Besides, many other reasons including economic setting and social security system may contribute to this difference.

Factors such as age (25), ethnicity (26), disease duration, disease activity (27, 28), physical function (28, 29), and quality of life (28) were reported to significantly affect work outcomes in other studies. Disease duration, BASFI and ASQoL were found to be related to work outcomes in this study, which concurs with studies done in Western (27, 29) and Asia populations (28). The current study showed no significant difference was found between disease activity (such as BASDAI or ASDAS) and work outcomes, which was inconsistent with other studies (27, 28) and the reason may be that only patients with BASDAI ≥4 were included in this study. However, controlling disease activity is still of importance to improve work outcomes in AS patients.

This study has limitations such as observational design which is unable to determine causality between factors and work outcomes. Secondly, the sample of this study was relatively small and only patients with high disease activity were included. In addition, the follow-up was restricted to 6 months and long-term effect of adalimumab on work outcome and cost was not reported in this study. Further study with larger samples and longer follow-up should be conducted in order to explore the long-term effect of biological therapy on cost and work outcomes in SpA and AS patients.

In conclusion, disease burden of AS patients with adalimumab therapy was huge in China. After 6 months' adalimumab therapy, improvement of disease activity, physical function, QoL and work outcomes was observed. Long disease duration, poor physical function and low quality of life were found to be related to poor work outcomes. Improvement of the social insurance system, early diagnosis and patient education would help to alleviate work disability in AS patients.

## Data Availability Statement

The original contributions presented in the study are included in the article/supplementary materials, further inquiries can be directed to the corresponding author/s.

## Ethics Statement

The studies involving human participants were reviewed and approved by the ethics committee of third affiliated hospital of Sun Yet-sen University. The patients/participants provided their written informed consent to participate in this study.

## Author Contributions

JG: had full access to all of the data in the study and takes responsibility for the integrity of the data and the accuracy of the data analysis. JG, LT, and YX: study concept, design, and drafting the manuscript. All authors: acquisition, analysis, or interpretation of data, critical revision of the manuscript for important intellectual content, and final approval of the article.

## Conflict of Interest

The authors declare that the research was conducted in the absence of any commercial or financial relationships that could be construed as a potential conflict of interest.
